# Supplementary material for: Assessing Spatial Distribution of Multicellular Self-Assembly Enables the Prediction of Phenotypic Heterogeneity in Glioblastoma
Source: Cancers (Basel). 2022 Nov 30;14(23):5910. doi: 10.3390/cancers14235910 (PMC9737258; doi:10.3390/cancers14235910)
Supplement: Supplementary file 1 [file cancers-14-05910-s001.zip › cancers-2010857-supplementary.pdf]

Supplementary Information

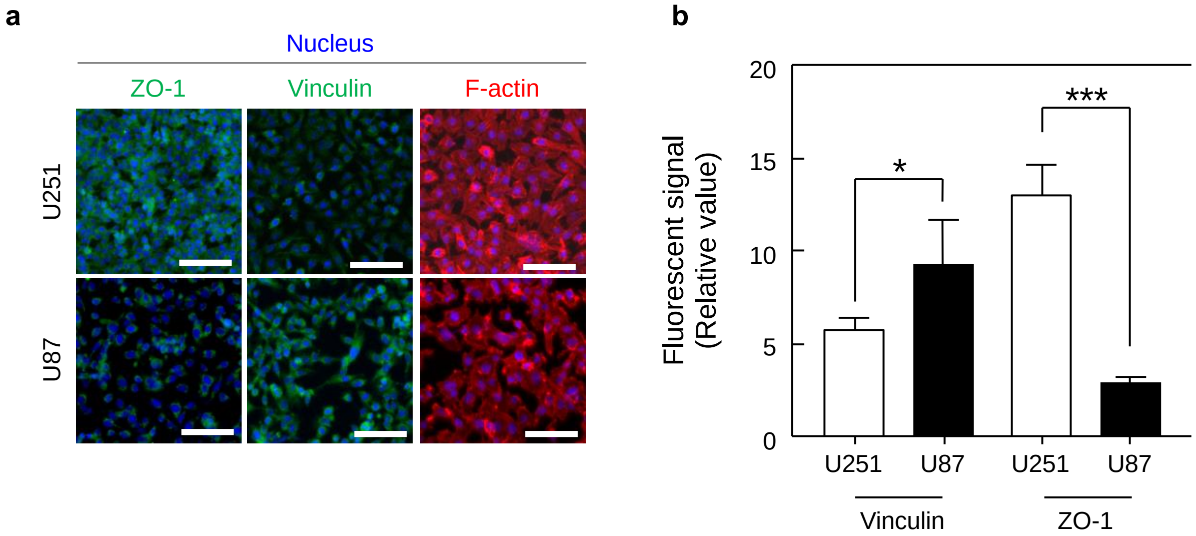

Figure S1. Cell-cell and cell-substrate interaction of U251 and U87 cell lines. (**a**) Representative fluorescent images for ZO-1 (cell-cell affinity) and Vinculin (cell-substrate interaction) expression on 2D. (**b)** Quantitative analysis for fluorescent signals from images. Statistical analysis: * $p < 0.05$, *** $p < 0.0001$ by Student's test.

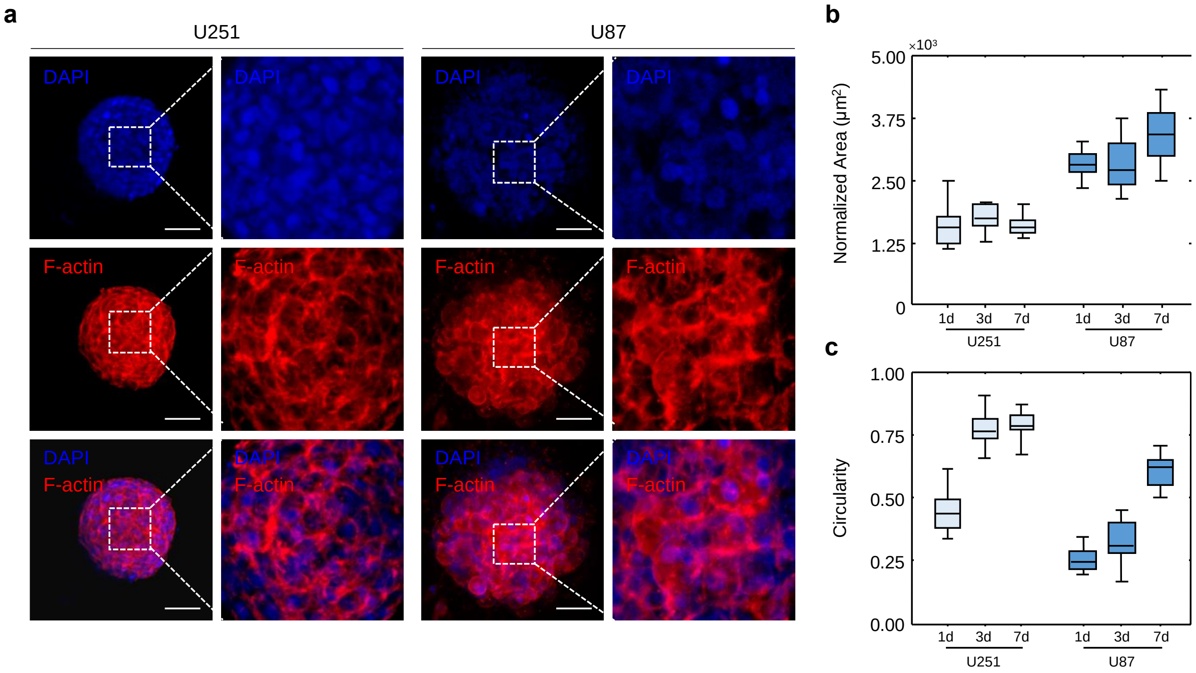

Figure S2. Structural characterization of U251 and U87 glioblastoma cell line features in 3D. (**a**) Fluorescent images for morphological comparison between U251 MAs and U87 MAs. Red: F-actin, Blue: Nucleus. Scale bar: 50 μm. Parametric analysis of morphologic features in glioblastoma MAs by measuring (**b**) the normalized area and (**c**) circularity of MAs.

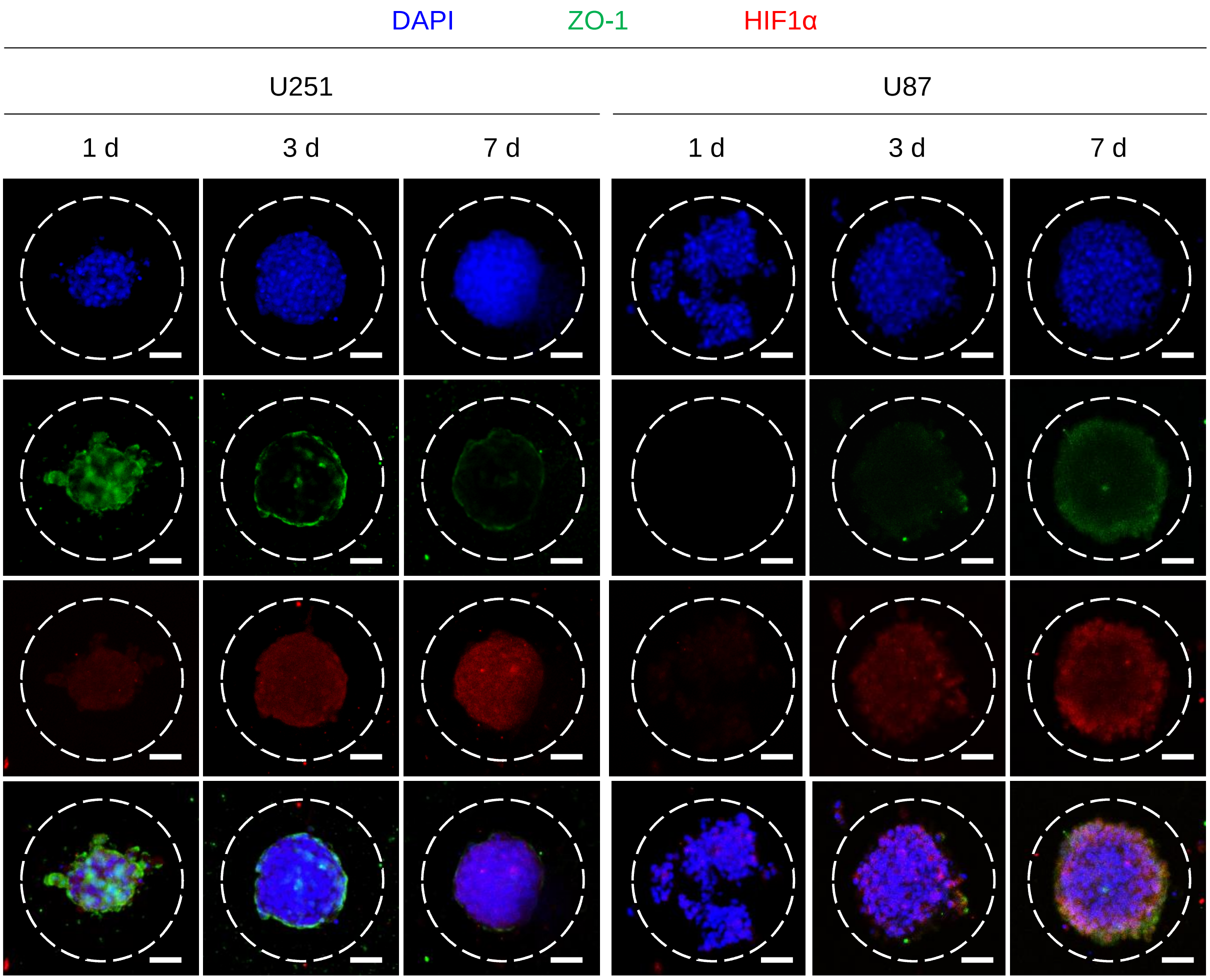

Figure S3. Immunofluorescent images of the ZO-1 and HIF1α expression in U251 MAs and U87 MAs. Dotted-line indicates the boundary of the single microwell. Scale bar: 50 μm.

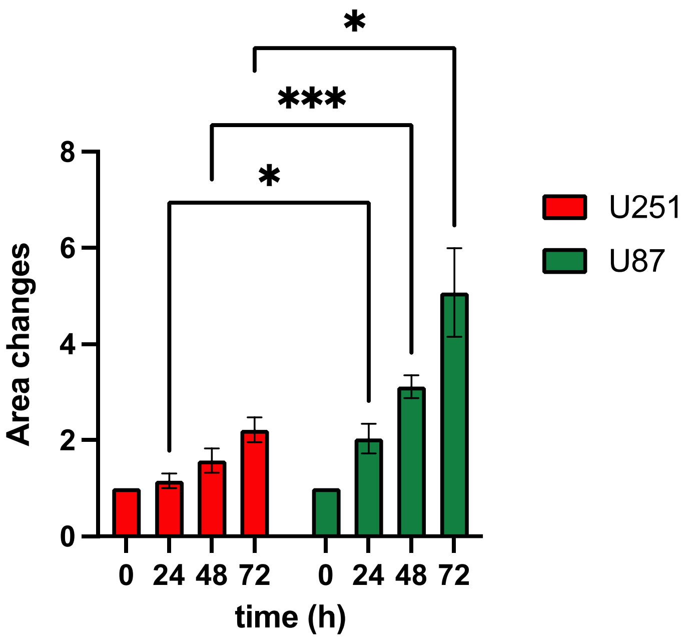

Figure S4. Quantification of U251-U87 MAs invasion. Statistical analysis: * $p < 0.05$, *** $p < 0.0001$ by Two-way ANOVA.

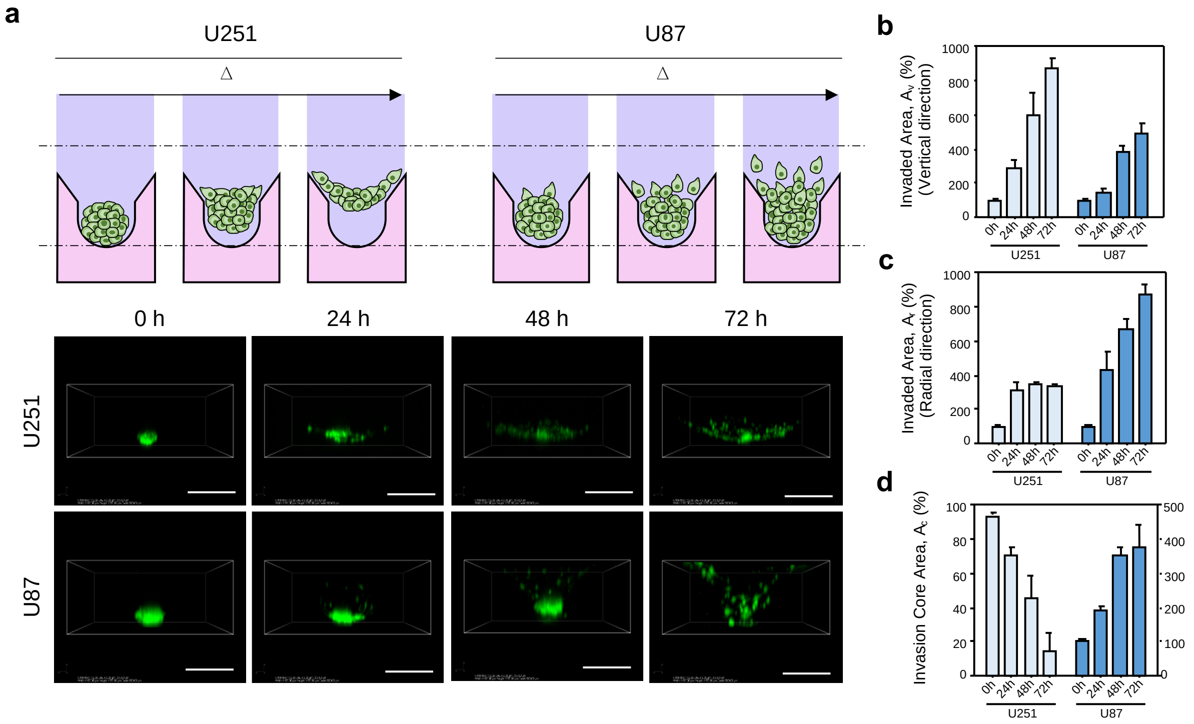

Figure S5. 3D Invasion assay and the quantification of the invasiveness for U251 MAs and U87 MAs. (**a**) Time-lapse invasion of U251 MAs and U87 MAs for 72 hrs. Quantification of glioblastoma MAs (**b**) in vertical direction, (**c**) in radial direction, and (**d**) changes in invasion core area.

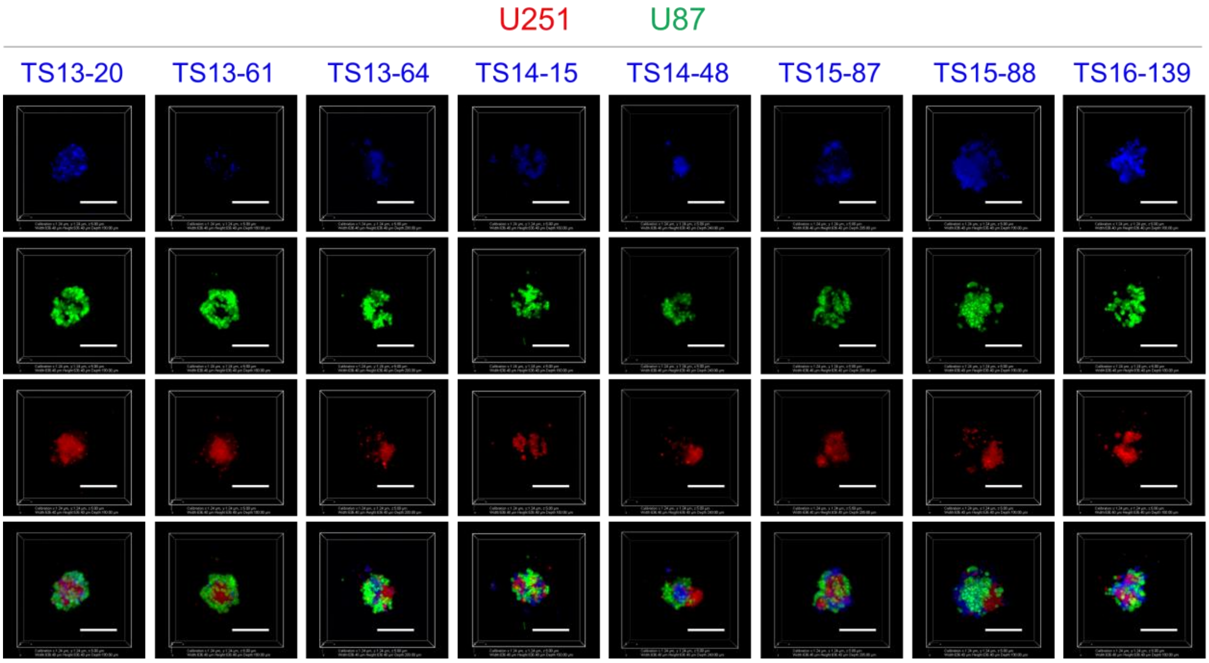

Figure S6. Heteroculture of patient-derived glioblastoma cells (pdGCs) in comparison to the U251 and U87 glioblastoma cell lines. Eight pdGCs labeled with CellTracker Blue were aggregated with U251 (red) and U87 (green) glioblastoma cells for forming the MAs.: TS13-20, TS13-61, TS13-64, TS14-15, TS15-87, TS15-88 and TS16-139. Scale bar: 200 μm.

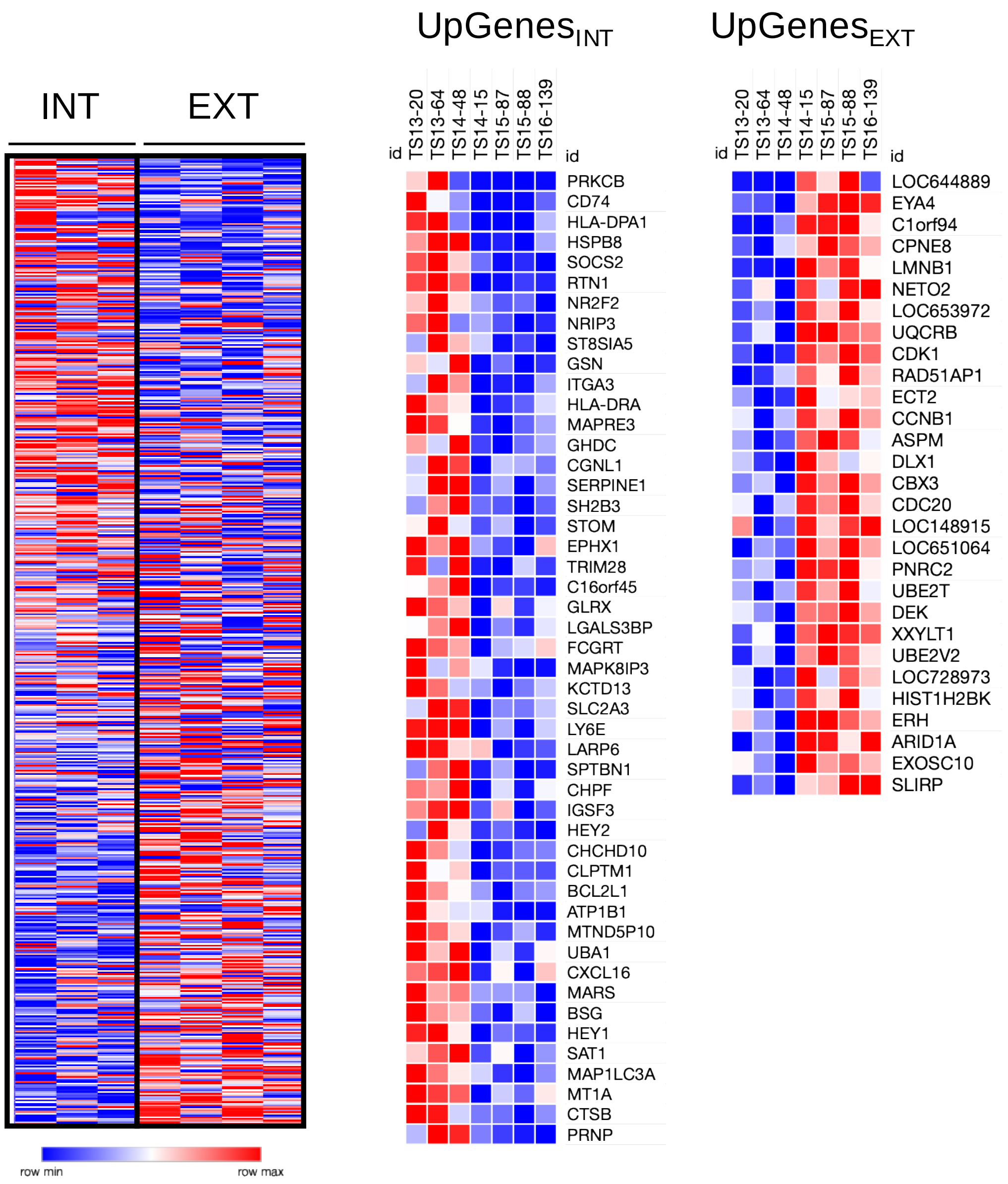

Figure S7. Hierarchical clustering of genes differentially expressed by internal and external pdGC types. Heatmaps of the unregulated gene expression levels of internal and external pdGC types.

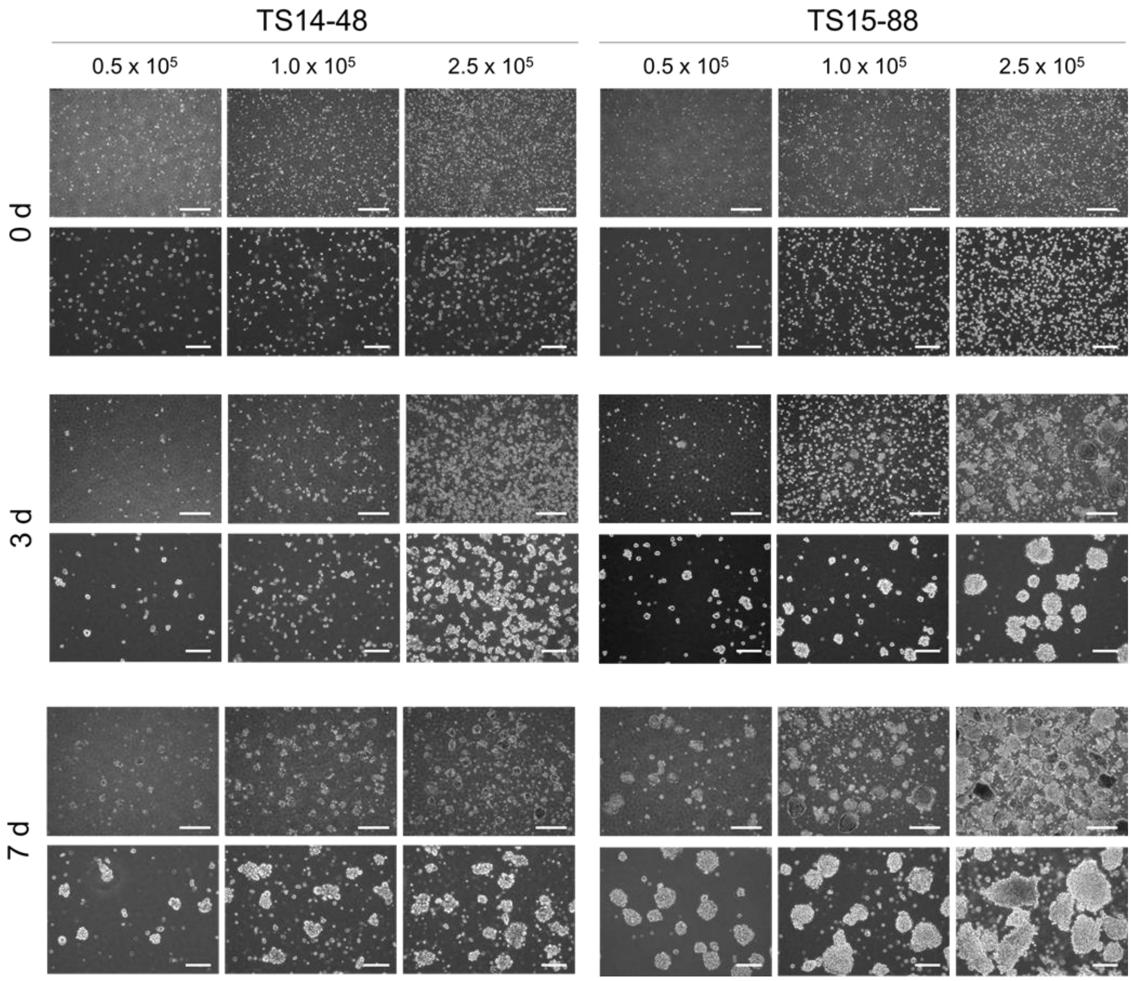

Figure S8. Representative images of pdGCs growth at the different cell density: 0.5×10^5, 1.0×10^5 and 2.5×10^5. The images were taken at 0, 3 and 7 days. Scale bar: 200 µm.

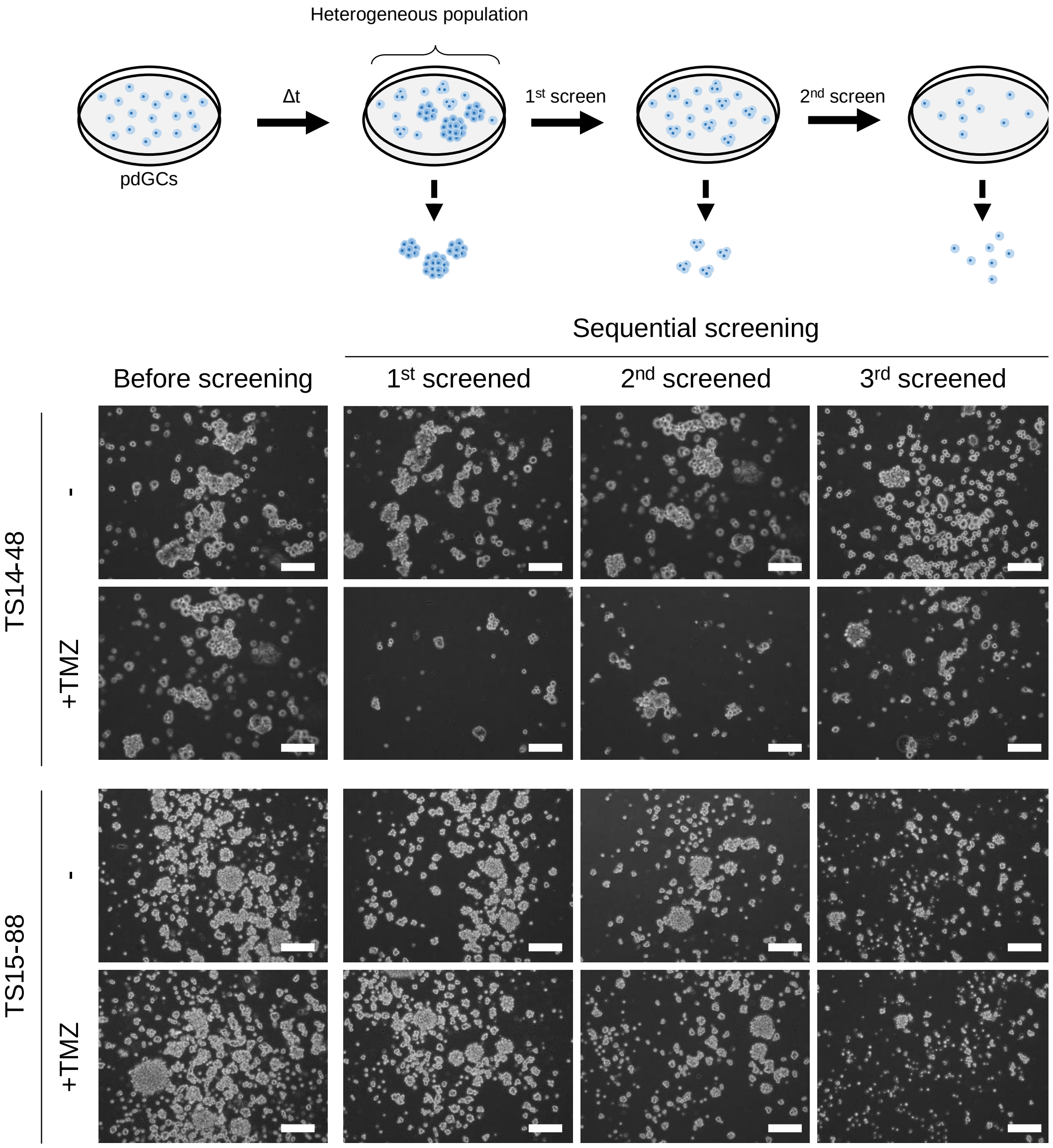

Figure S9. Heterogeneous drug response within a pdGC population. Screening heterogeneous features of subpopulations from a single pdGC. Representative images for cell viability in response to the TMZ treatment of screened subpopulations from a single pdGCs.

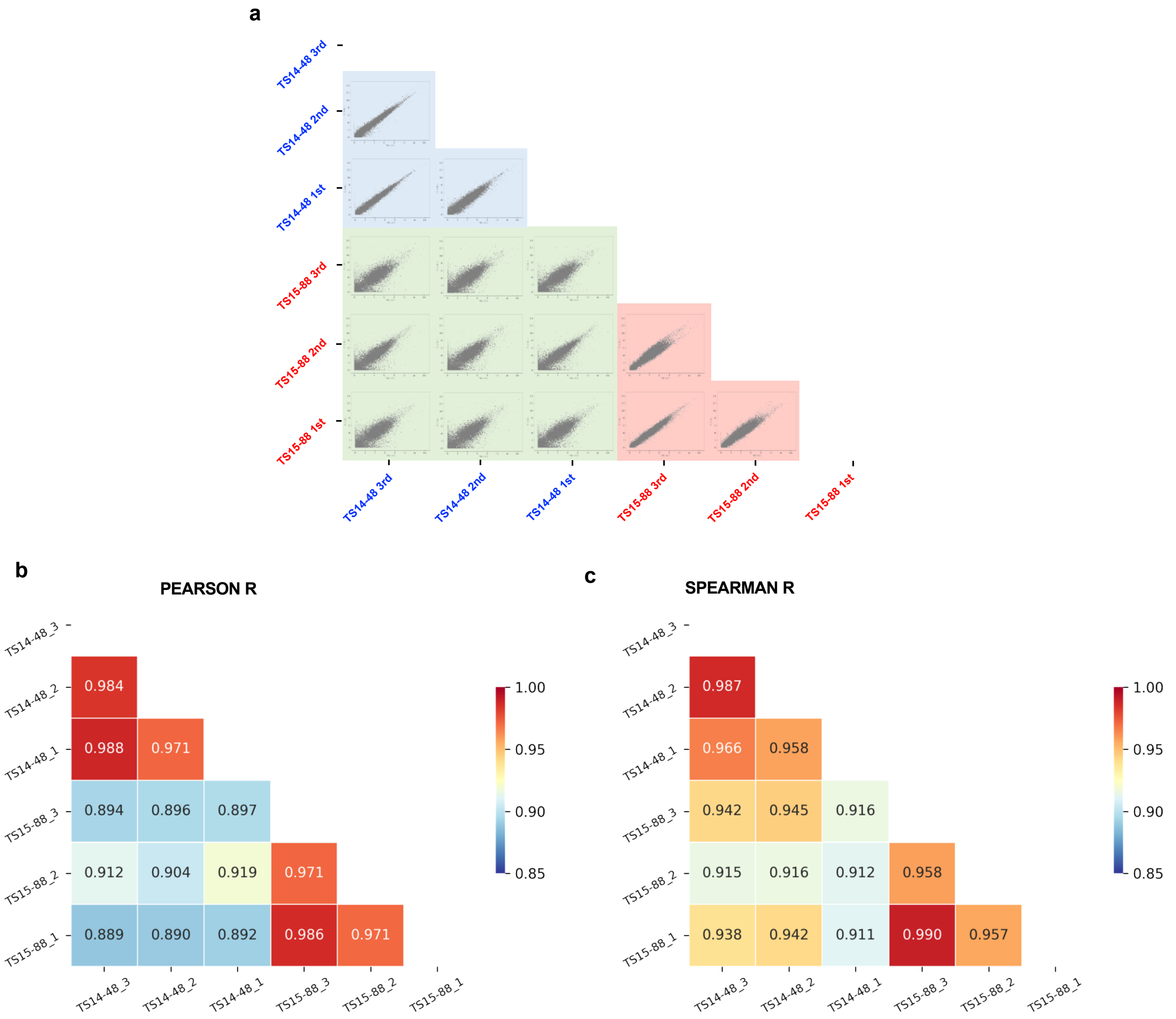

Figure S10. (**a**) Scatter plots of correlations for sequentially screened TS14-48 and TS15-88 subpopulations. (**b**) Pearson's and (**c**) Spearman's correlation analysis for sequentially screened TS14-48 and TS15-88 subpopulations.

Table S1. Statistics for quantification of pdGC invasion in Figure 4c. Statistical significance: ** $p < 0.01$, *** $p < 0.001$, **** $p < 0.0001$. ns: no significance.

Tukey's multiple comparisons test	Mean Diff.	95.00% CI of diff.	Below threshold?	Summary	Adjusted *p* Value
TS13-20 vs. TS13-61	1.432	0.5692 to 2.294	Yes	****	<0.0001
TS13-20 vs. TS13-64	-0.7973	-1.660 to 0.06533	No	ns	0.0904
TS13-20 vs. TS14-15	2.176	1.313 to 3.038	Yes	****	<0.0001
TS13-20 vs. TS14-48	2.010	1.147 to 2.872	Yes	****	<0.0001
TS13-20 vs. TS15-87	0.5737	-0.2889 to 1.436	No	ns	0.4361
TS13-20 vs. TS15-88	-1.155	-2.018 to -0.2928	Yes	**	0.0021
TS13-20 vs. TS16-139	2.185	1.322 to 3.048	Yes	****	<0.0001
TS13-61 vs. TS13-64	-2.229	-3.092 to -1.366	Yes	****	<0.0001
TS13-61 vs. TS14-15	0.7438	-0.1188 to 1.606	No	ns	0.1409
TS13-61 vs. TS14-48	0.5778	-0.2849 to 1.440	No	ns	0.4269
TS13-61 vs. TS15-87	-0.8581	-1.721 to 0.004489	No	ns	0.0522
TS13-61 vs. TS15-88	-2.587	-3.450 to -1.725	Yes	****	<0.0001
TS13-61 vs. TS16-139	0.7532	-0.1094 to 1.616	No	ns	0.1307
TS13-64 vs. TS14-15	2.973	2.110 to 3.836	Yes	****	<0.0001
TS13-64 vs. TS14-48	2.807	1.944 to 3.669	Yes	****	<0.0001
TS13-64 vs. TS15-87	1.371	0.5084 to 2.234	Yes	***	0.0001
TS13-64 vs. TS15-88	-0.3581	-1.221 to 0.5045	No	ns	0.8953
TS13-64 vs. TS16-139	2.982	2.120 to 3.845	Yes	****	<0.0001
TS14-15 vs. TS14-48	-0.1661	-1.029 to 0.6965	No	ns	0.9987
TS14-15 vs. TS15-87	-1.602	-2.465 to -0.7394	Yes	****	<0.0001
TS14-15 vs. TS15-88	-3.331	-4.194 to -2.468	Yes	****	<0.0001
TS14-15 vs. TS16-139	0.009364	-0.8532 to 0.8720	No	ns	>0.9999
TS14-48 vs. TS15-87	-1.436	-2.298 to -0.5733	Yes	****	<0.0001
TS14-48 vs. TS15-88	-3.165	-4.028 to -2.302	Yes	****	<0.0001
TS14-48 vs. TS16-139	0.1755	-0.6872 to 1.038	No	ns	0.9982
TS15-87 vs. TS15-88	-1.729	-2.592 to -0.8665	Yes	****	<0.0001
TS15-87 vs. TS16-139	1.611	0.7487 to 2.474	Yes	****	<0.0001
TS15-88 vs. TS16-139	3.340	2.478 to 4.203	Yes	****	<0.0001
